# Novel broad spectrum virucidal molecules against enveloped viruses

**DOI:** 10.1371/journal.pone.0208333

**Published:** 2018-12-07

**Authors:** Valeria Cagno, Cristina Tintori, Andrea Civra, Roberta Cavalli, Marika Tiberi, Lorenzo Botta, Annalaura Brai, Giulio Poli, Caroline Tapparel, David Lembo, Maurizio Botta

**Affiliations:** 1 Laboratory of Molecular Virology and Antiviral Research, Department of Clinical and Biological Sciences, University of Torino, Orbassano, Torino, Italy; 2 Department of Molecular Microbiology, University of Geneva, Geneva, Switzerland; 3 Department of Biotechnology, Chemistry and Pharmacy, University of Siena, Siena, Italy; 4 Department of Drug Science and Technology, University of Torino, Torino, Italy; 5 Lead Discovery Siena S.r.l., Castelnuovo Berardenga, Siena, Italy; 6 Sbarro Institute for Cancer Research and Molecular Medicine, Center for Biotechnology, College of Science and Technology, Temple University, Philadelphia, PA, United States of America; Cornell University, UNITED STATES

## Abstract

Viral infections are an important cause of death worldwide. Unfortunately, there is still a lack of antiviral drugs or vaccines for a large number of viruses, and this represents a remarkable challenge particularly for emerging and re-emerging viruses. For this reason, the identification of broad spectrum antiviral compounds provides a valuable opportunity for developing efficient antiviral therapies. Here we report on a class of rhodanine and thiobarbituric derivatives displaying a broad spectrum antiviral activity against seven different enveloped viruses including an HSV-2 acyclovir resistant strain with favorable selectivity indexes. Due to their selective action on enveloped viruses and to their lipid oxidation ability, we hypothesize a mechanism on the viral envelope that affects the fluidity of the lipid bilayer, thus compromising the efficiency of virus-cell fusion and preventing viral entry.

## Introduction

Viral infections are one of the ten leading causes of death worldwide [1]. Nowadays, although effective antiviral strategies have been successfully developed for some important pathogens such as HIV and HCV, antiviral drugs or vaccines are still missing for the majority of viruses. As an example, no effective antiviral strategies are yet available for viruses causing chronic infections such as HBV [2], as well as for tropical viruses like dengue virus that is causing 390 million infections per year [3]. A particular interest is addressed to the recent epidemics of Ebola in Africa and Zika (ZIKV) in South America, for which, despite the huge effort to find antivirals, the research community was not able to execute in time an efficient antiviral plan.

The majority of emerging or re-emerging viruses are zoonoses, and it has been demonstrated that the passage among different species is easier for enveloped viruses in comparison with non-enveloped viruses [4, 5]. It is estimated that there are approximately 500,000 unknown mammalian viruses in animal reservoirs [6]. In this scenario, the possibility to find antiviral molecules directed against viral envelopes is of particular interest in order to identify broad spectrum antiviral drugs [7].

In our previous papers we reported some rhodanine and aminothiazolone derivatives endowed with nanomolar activities against HIV-1 infected cells [8–10]. In a recent work, we showed that our compounds were also active against HSV-1/2, while they were completely ineffective against HPV, a nonenveloped virus [11]. These results suggested that the mode of action of our molecules could involve the viral envelope. Compounds acting on viral envelopes have been previously described: RAFI compounds inhibit a broad range of enveloped viruses by inserting into viral envelopes and altering the fusion kinetics in the hemifusion stalk due to their inverted cone structure [12]. Liposomes extracting cholesterol from viral envelopes have been reported to inhibit HIV, HCV and HBV [13]. Virolytic antiviral peptides derived from mastoparan were shown to inhibit different enveloped viruses acting on the envelope and causing its detachment from the viral core [14]. The selectivity of these strategies is based on the fact that viral envelopes are static and characterized by an absence of repair mechanisms, in contrast with the biogenic membranes of the cells that are endowed with plenty of tools to repair membrane damage or alteration [15]. Furthermore, since envelope lipids are derived from host cells, it is more complicated to the virus to develop resistance to these compounds if compared to those targeting the classical viral components; for this reason they are extremely promising targets also for viruses with a high mutation rate.

Therefore, we investigated the activity on HSV-2 of a new series of compounds. After having selected the most potent derivative of the series, we verified its broad spectrum activity against an HSV-2 acyclovir-resistant strain and other six important enveloped pathogens such as ZIKV, influenza A virus (IAV) and respiratory syncytial virus (RSV), amongst the others, and we confirmed its complete inactivity against three non-enveloped viruses. Finally, we investigated its mechanism of action, identifying a lipid oxidizing activity and the impairment of viral entry of HSV-2.

## Results and discussion

### Chemistry

**Fig 1:**
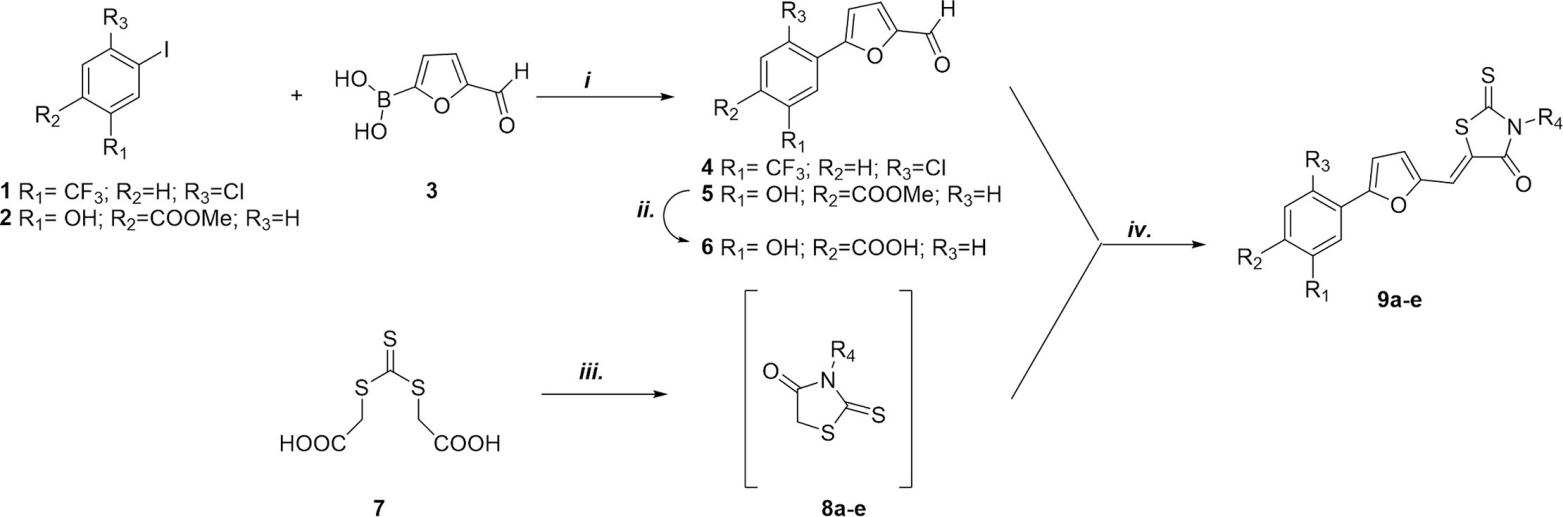
Synthesis of substituted rhodanine derivatives 9 a–e: *i*. Pd(PPh_3_)_2_Cl_2_, Na_2_CO_3_, DMF/EtOH, RT, 1h; *ii*. 1N NaOH (aq), MeOH/THF, reflux; *iii*. DME, Et_3_N, MW (300 W), 90°C, 10 min. *iv*. aldehyde 4 or 6, DME, MW (300 W), 110°C, 5 min.

The compounds were synthesized using the one pot two step approach previously generated by us for the synthesis of rhodanine derivatives. According to Fig 1, aldehydes **4–6** were obtained through Suzuki reaction between commercially available iodides **1** or **2** and 5-formyl-2-furanylboronic acid **3**. Basic hydrolysis of ester **5** provided the corresponding acid **6**. Nucleophilic substitution between trithiocarbonate and the opportune primary amine led to the rhodanine intermediates **8a-e**, which were converted into the final compounds **9a-e** by Knoevenagel condensation with aldehyde **4** or **6**.

**Fig 2:**
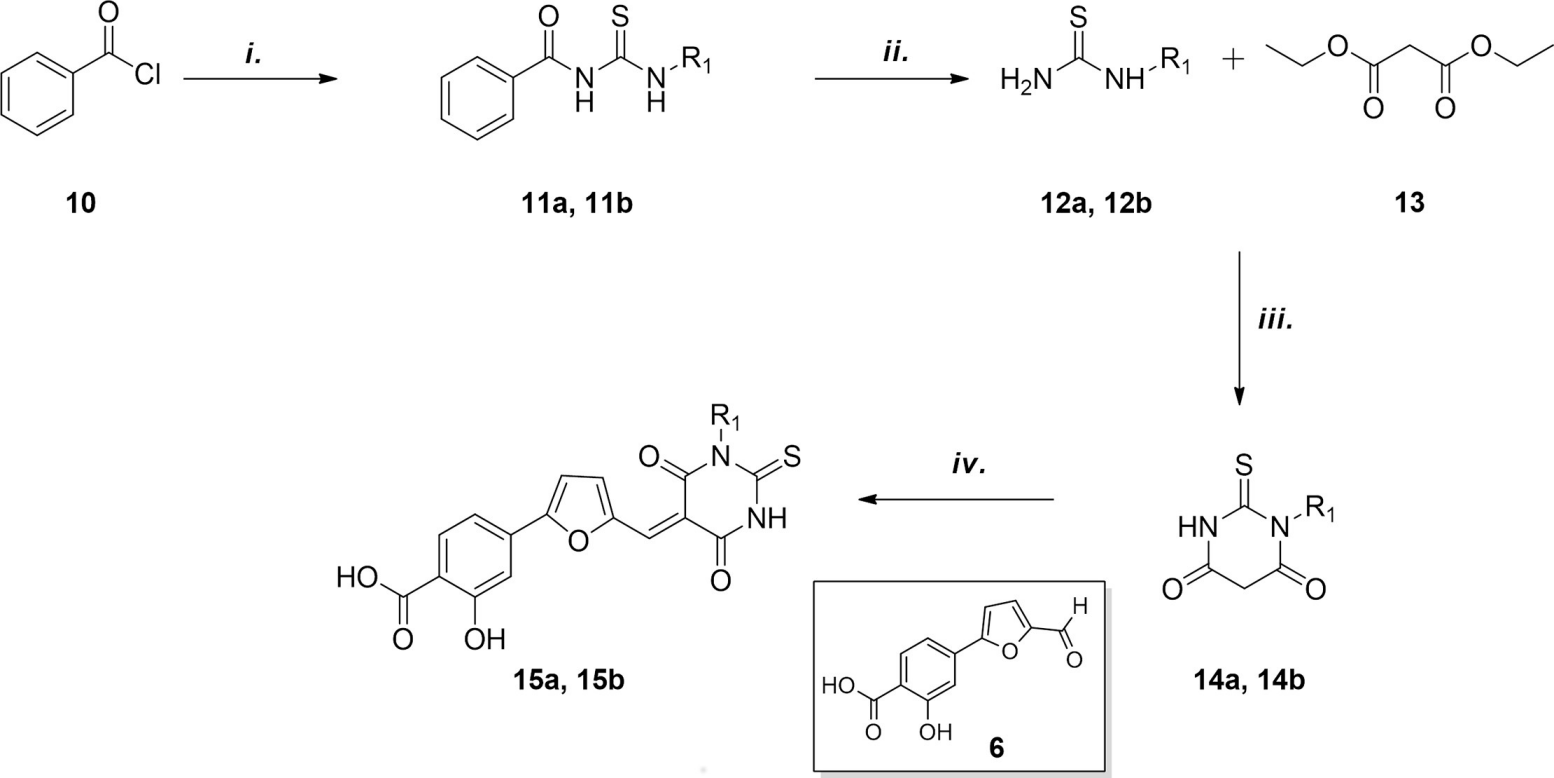
Synthesis of monosubstituted thiobarbituric compounds **15a** and **15b**: i. a) NH_4_SCN, (CH_3_)_2_CO, 60°C MW, min, b) opportune amine (CH_3_)_2_CO, 60°C MW, ii. NH_2_NH_2_; iii. Na^0^, EtOH, reflux, iv. HCl, EtOH, 70°C.

Thiobarbituric derivatives were synthesized as shown in Fig 2. Reaction between benzoyl chloride **10**, ammonium thiocyanate and the opportune amine led to intermediates **11a** and **11b;** consequent hydrazinolisis and coupling reaction with diethylmalonate **13** in basic conditions furnished monoalkylated thiobarbituric derivatives **14a** and **14b**. Finally Knoevenagel condensation with aldehyde **6** in refluxing acidic EtOH led to compounds **15a** and **15b**.

### Evaluation of antiviral activity against HSV-2

**Fig 3:**
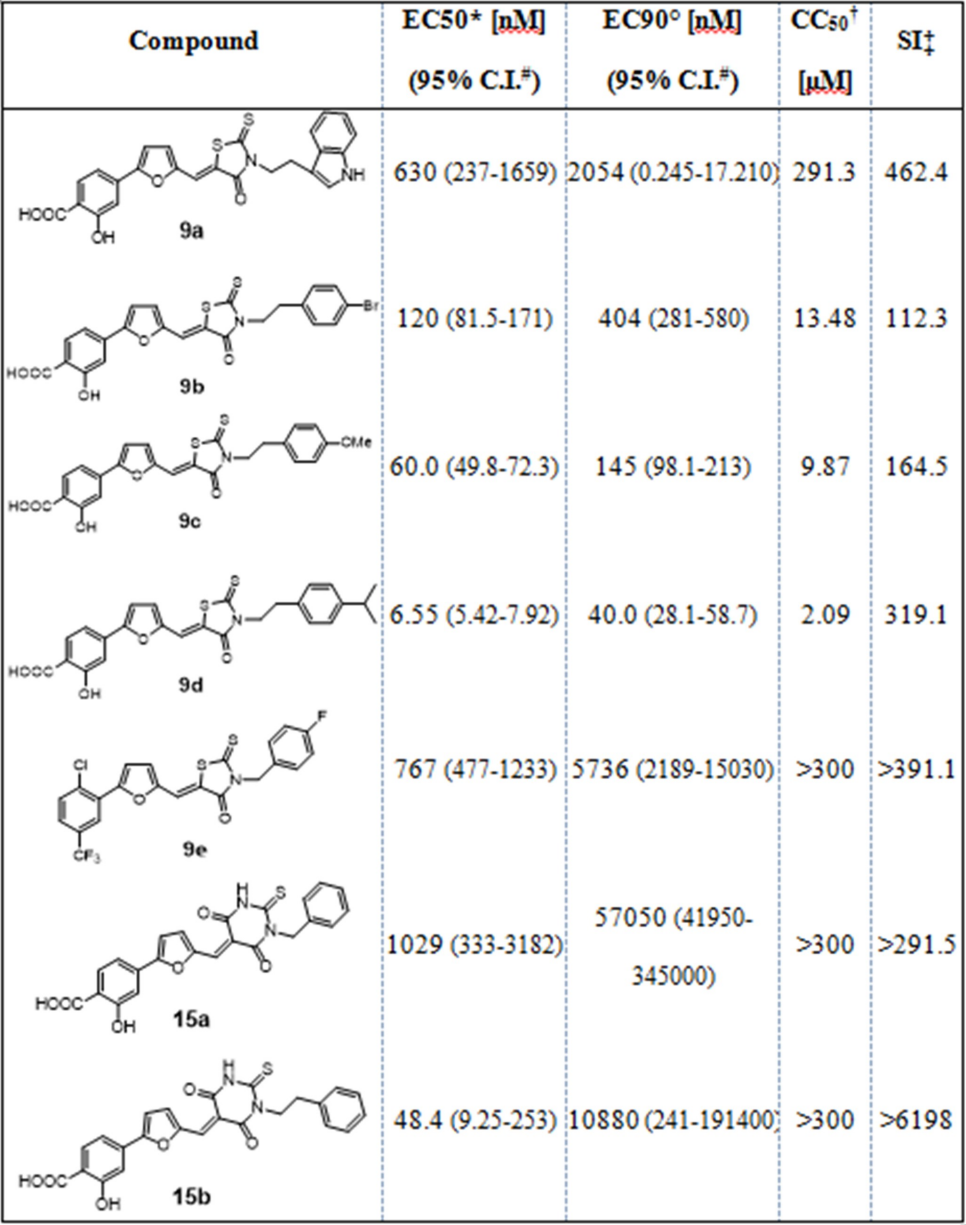
Antiviral activity against HSV-2. *EC_50_ half maximal effective concentration; °EC_90_ 90% effective concentration; **#**C.I. confidence interval; †CC_50_ half maximal cytotoxic concentration; ‡SI selectivity index; n.a. not assessable. 9d n = 5, other compounds n = 2.

Having proved that rhodanine derivatives belonging to this series of compounds are potential microbicide active against HIV-1 and HSV-1/2 [11], we further investigated the inhibitory activity against HSV-2 of the novel derivatives bearing both rhodanine and thiobarbituric scaffolds. The compounds were tested with a plaquing efficiency assay, preincubating with the virus for 1 h at 37°C and subsequently adding the mixtures on cells. All the compounds exhibited potent inhibitory activities at non-cytotoxic doses, as demonstrated by the high selectivity indexes (Fig 3). Compound **9d**, which showed the most promising antiviral activity, was subjected to further investigations. The toxicity of compound **9d** was evaluated at different time points on Vero cells, and also after 96h exposure the CC_50_ was evaluated to be 2.09 μM confirming the favorable selectivity index (S1 Fig).

### Compound 9d has a broad antiviral activity against enveloped viruses

Due to the lipophilic nature of the compound and the results obtained in previous works, in which derivatives of the same series proved to be effective against HIV and HSV [11], we investigated the spectrum of antiviral activity of **9d** against different enveloped viruses (i.e. HSV-1, HCMV, RSV, ZIKV, IAV, VSV) and non-enveloped viruses (i.e. Ad5, HPV and HRoV). As reported in Table 1, the compound showed a potent antiviral activity against all enveloped viruses tested at not cytotoxic doses, while it was found to be inactive against non-enveloped viruses, suggesting a possible action on the viral envelope. Interestingly, compound **9d** retained its antiviral activity also against HSV-2 acyclovir resistant strain, suggesting a different mechanism of action.

**Table 1:** Spectrum of antiviral activity of compound 9d.

Type of virus	Virus	EC50* [nM] (95% C.I.^#^)	EC90° [nM] (95% C.I.^#^)	CC_50_^†^ [μM]	SI‡
Enveloped	HSV-2^a^	1.60 (1.54–1.66)	24.3 (18.7–31.5)	2.09	1306
Enveloped	HSV-1	2.18 (0.91–5.20)	35.1 (6.70–184)	2.09	958.7
Enveloped	HCMV	4.22 (2.89–6.16)	31.3 (13.3–76.7)	5.24	1241
Enveloped	RSV	54.8 (48.2–62.2)	221 (162–301)	17.04	291.8
Enveloped	ZIKAV	69.9 (57.3–85.3)	147 (81–240)	8.85	126.6
Enveloped	IAV	66.1 (54.7–80.1)	301.2 (201.3–470)	135.7	2053
Enveloped	VSV	13.2 (9.58–18.3)	117 (57.2–239)	24.84	1881
Non enveloped	HPV-16	n.a.	n.a.	94.23	n.a.
Non enveloped	Ad5	n.a.	n.a.	94.23	n.a.
Non enveloped	HRoV	n.a.	n.a.	156.1	n.a.

^a^Acyclovir resistant strain

*EC_50_ half maximal effective concentration

°EC_90_ 90% effective concentration

**#**C.I. confidence interval

†CC_50_ half maximal cytotoxic concentration

‡SI selectivity index; n.a. not assessable. n = 2.

### The antiviral activity is directed to the virus rather than the cell, but is maintained in post treatment

**Fig 4:**
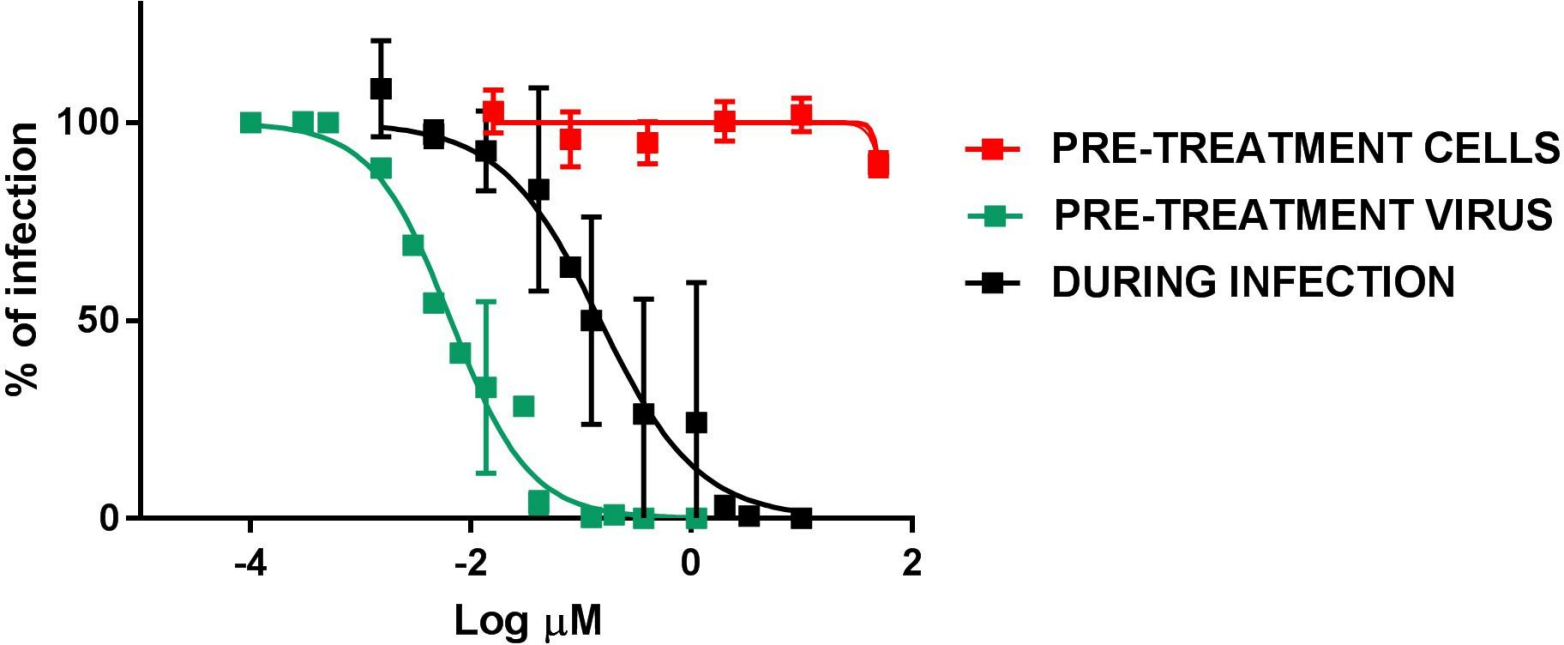
Time of addition assays. Compound **9d** was added on cells 2 h before infection (PRE-TREATMENT CELLS) and subsequently the inocula were removed and cells were infected or compound and virus were added simultaneously on cells (DURING INFECTION) or after 1 h pre-incubation between the virus and the compound (PRE-TREATMENT VIRUS). Cells were fixed and stained 24 h later and plaques were counted. The percentage of infection was calculated comparing treated wells to wells treated with equal volumes of solvent. n = 3.

We performed assays aimed at better understanding the mechanism of action. In the pretreatment assay compound **9d** was added on cells before the virus in order to determine if the antiviral activity was due to an interaction with the cells. Moreover, an assay was conducted adding virus and compound on cells without the preincubation performed in previous assays. The results showed in Fig 4 demonstrate that the antiviral activity of **9d** is not due to an interaction with cells, since the compound was not active in pre-treatment assays. On the contrary, we observed a significant reduction of activity in the during-infection assay (EC_50_ = 0.147 μM), if compared to the pre-incubation assay (EC_50_ = 6.55 nM) suggesting that the loss of activity could be related to a competition between the viral envelope and the cell membrane for the insertion and activity of the compound.

**Fig 5:**
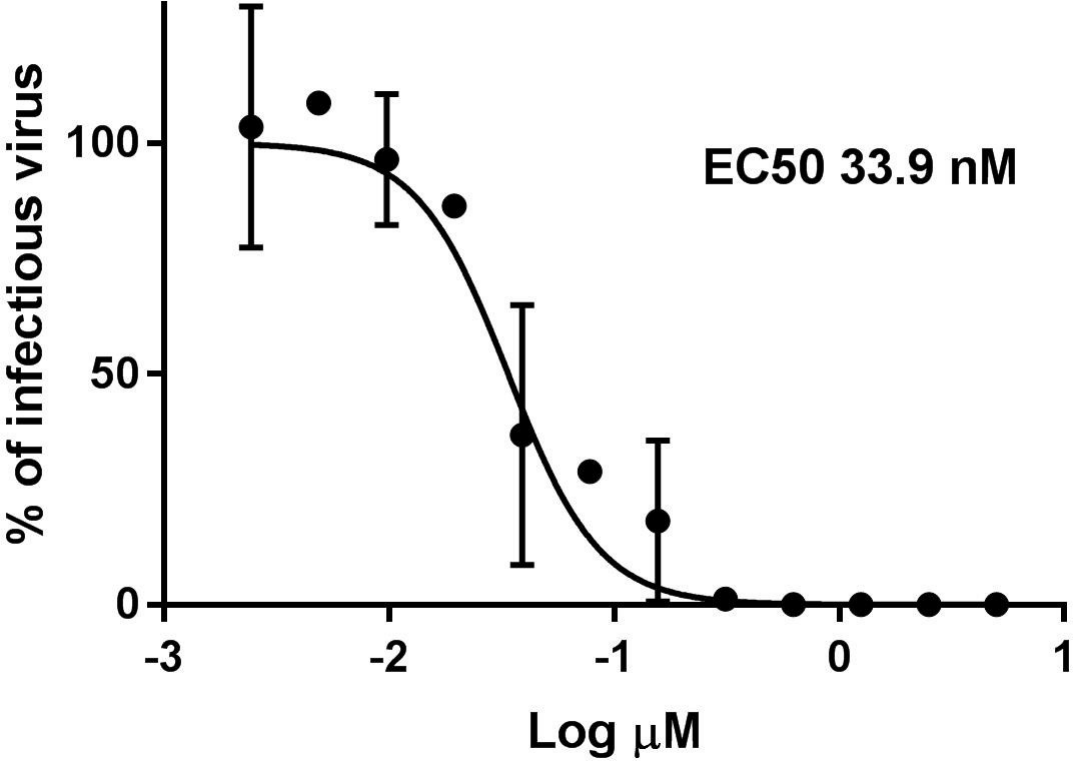
Viral yield reduction. Cells were infected with HSV-2 (MOI 0.01) and after the removal of inoculum they were treated with increasing concentrations of compound. At 24 hpi when the untreated wells showed extensive cytopathology, supernatants and cells were harvested and subsequently titrated with plaque assay. The percentage of infection was calculated comparing viral titer in treated wells and wells treated with equal volumes of solvent. n = 4.

We then investigated whether compound **9d** was able to inhibit multiple cycles of infection, in viral yield reduction assays, when added post infection. As shown in Fig 5, in this condition the compound retained a good antiviral activity, suggesting possible therapeutic uses.

### Virucidal activity

**Fig 6:**
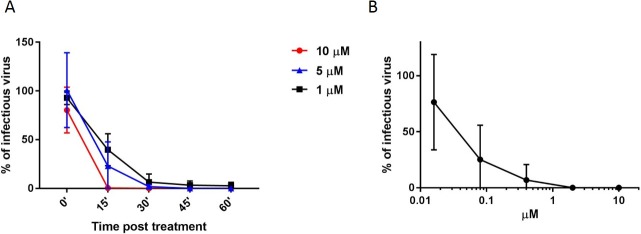
Virucidal activity. A) 10^5^ pfu of HSV-2 were incubated for 0, 15’, 30’, 45’ or 60’ at 37°C with 10 μM, 5 μM or 1 μM of compound and subsequently titrated on Vero cells. B) 10^5^ pfu of HSV-2 were incubated with different doses of compounds 9d for 1h and subsequently titrated on Vero cells. Viral titers were calculated at dilutions of compounds at which, in plaquing efficiency assay, did not show any inhibitory activity. The percentage of infection was calculated comparing viral titer in treated wells and wells treated with equal volumes of solvent. Results show the mean and SD or square roots of the sum of squares, n = 6 for 60’ and n = 2 for other conditions.

To further elucidate the mechanism of action we performed a virucidal assay in which **9d** was incubated with the virus at 10 μM 5μM or 1μM concentration for different times (Fig 6A) or for 1h with serial dilutions of compound (Fig 6B); subsequently, the mixture was titrated on cells and the viral titer was evaluated at dilutions at which the compound concentration was known not to be active in plaquing efficiency assays. In these conditions it was possible to observe that the viral infectivity in presence of the compound was decreased at 15’ post treatment and completely abrogated from 30’ on when the virus was exposed to 10 μM of compound, and at lower doses (Fig 6A). While after 1h of exposure, the EC50 was 37.3 nM (Fig 6B). The irreversibility of the mechanism was also tested with an assay in which the compound was incubated with the virus for 1h and subsequently the mixture has been diluted in drug free medium for additional 1, 2, 3 or 4 hours before the addition on cells (S2 Fig). Also in this condition the infectivity was not regained further verifying the permanent virucidal activity.

**Fig 7:**
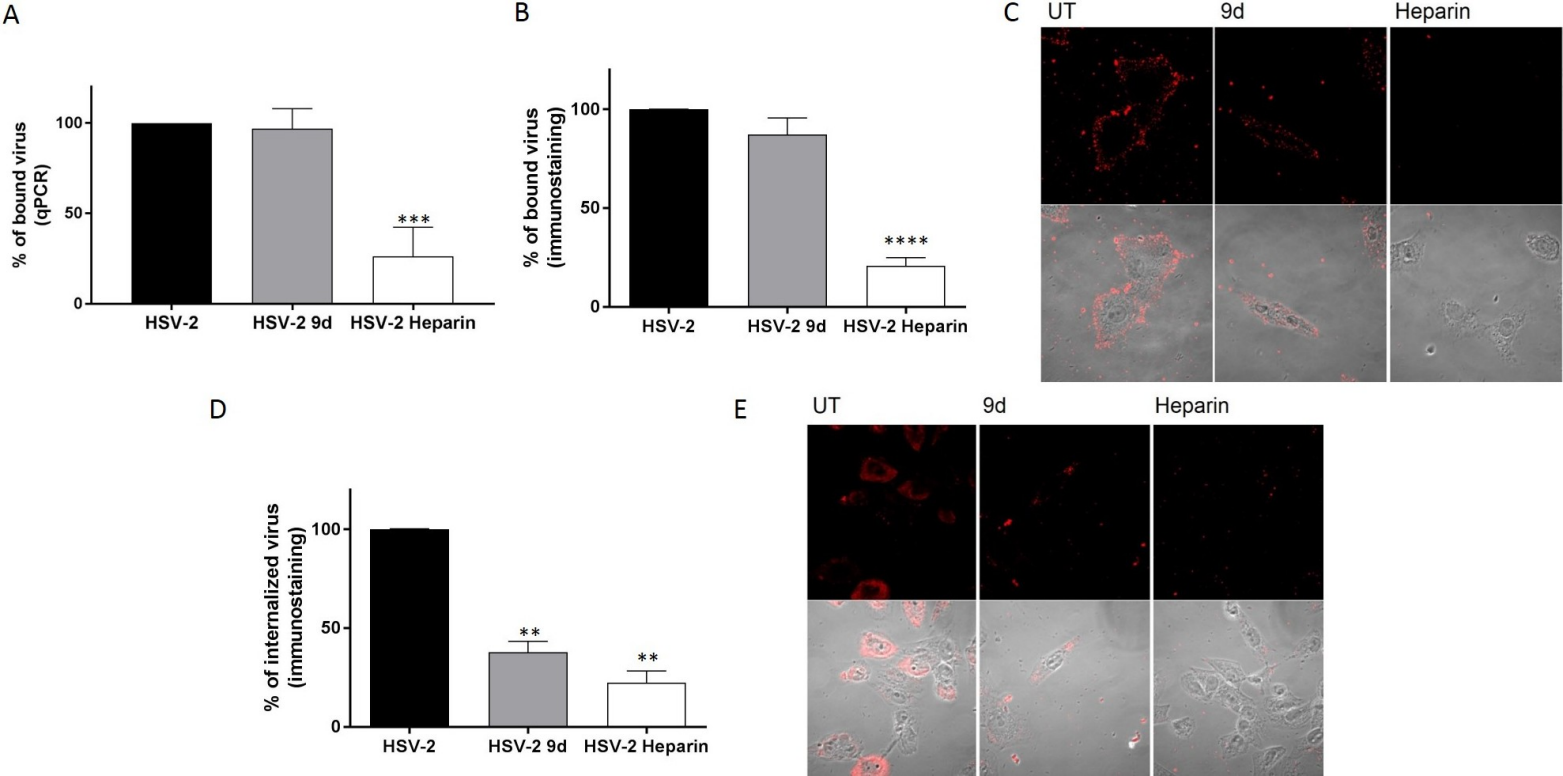
Binding and entry assays. (A-B-C) HSV-2 (MOI 10) was incubated for 1 h at 37°C with 10 μM of compound and subsequently added for 2 h on Vero cells at 4°C. Subsequently cells were subjected to qPCR (A) or immunostaining (B) or IF (C). (D-E) HSV-2 (MOI 10) was incubated for 1 h at 37°C with 10 μM of compound and subsequently added for 1 h on Vero cells at 4°C and then shifted at 37°C for 1.5 h (D) or 4 h (E) subsequently cells were fixed and subjected to ICC (D) or IF (E). UT = untreated ** p<0.001 *** p<0.0005 **** p<0.0001. n = 3.

Since the observed irreversible effect can be exerted at different stages of viral infection, we investigated more in detail if compound **9d** produced a physical disruption of the virus, thus preventing its attachment to the cells or a successive irreversible modification. We performed entry and binding assays, evaluating the amount of bound virus through immunostaining or qPCR. The results shown in Fig 7A, 7B and 7C, demonstrate that the compound is not altering viral binding capability, differently from heparin, a known attachment inhibitor [16]. On the contrary, when entry assays were performed, it was possible to observe a reduction in the amount of virus associated to the cells (Fig 7D). With this assay, the signal from the virus could be related to virus in the cytoplasm or blocked in the endosomes, since the treatment with acidic glycine is affecting only the virus at the surface of the cell. For this reason we performed also an in immunofluorescence (IF) in which it was possible to visualize that there is virus signal in discrete dots, possibly endosomes, while in the control the viral signal is diffused in the cytoplasm (Fig 7E), supporting the hypothesis that part of the virus could be blocked in endosomes due to an impaired fusion.

### In vitro determination of the compound peroxidative capability

**Fig 8:**
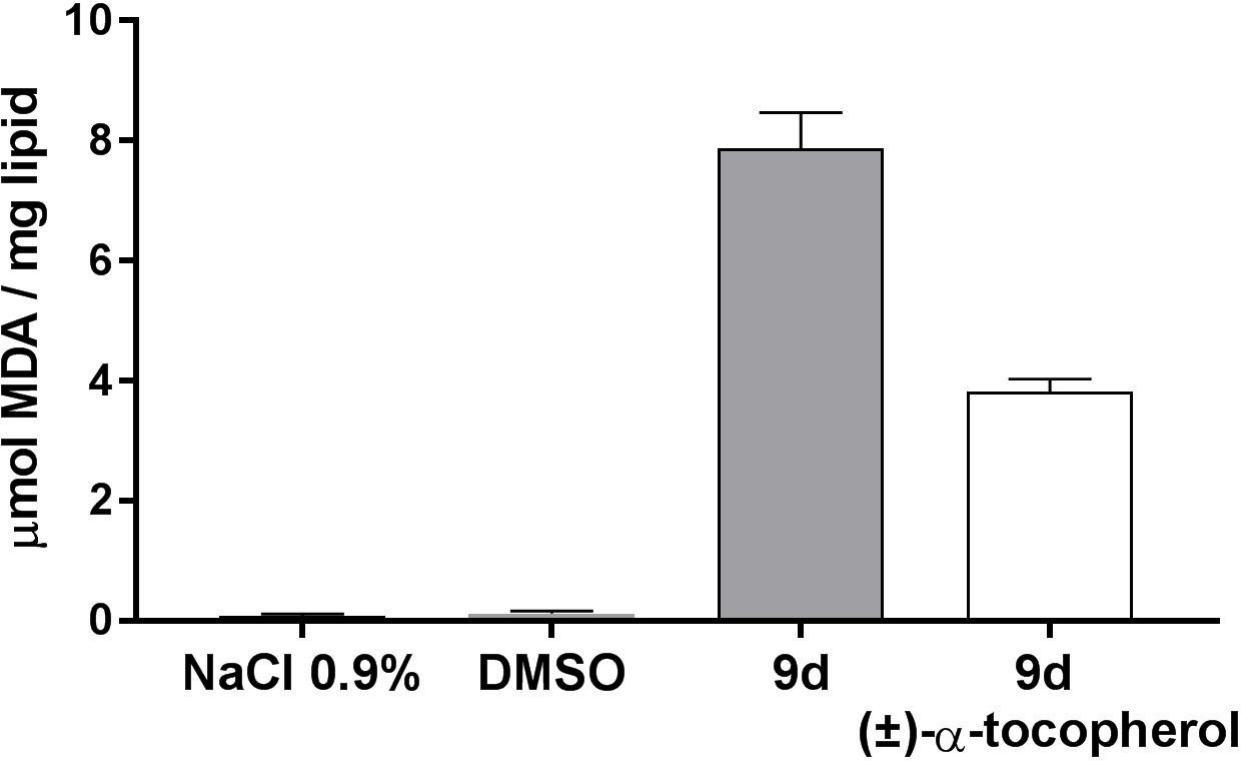
In vitro determination of lipid peroxidation capability. MDA (micromoles/mg) produced from the linoleic acid peroxidation after exposure to compound 9d in the presence and in the absence of (±)-α-tocopherol. ** p<0.01 NaCl 0.9% and DMSO are blank samples prepared without the addition of 9d and (±)-α-tocopherol. n = 3.

Due to the chemical structure of compound **9d** and its affinity for lipids, we envisioned that its mechanism of action might involve the peroxidation of viral phospholipids, which would lead to altered fusion properties of enveloped viruses. To assess this hypothesis, we carried out an in vitro lipoperoxidation assay based on the oxidation of linoleic acid as model lipid substrate, incorporated in a DPPC liposomes. The lipid peroxidation was evaluated by monitoring the production of the lipid degradation end-product malondialdehyde (MDA). After the exposure to compound **9d**, the linoleic acid underwent a remarkable lipid peroxidation in the liposome system (Fig 8). The results of the TBA assay suggest the peroxidant activity of the compound. The peroxidation capability of **9d** was further confirmed by the reduction of linoleic acid peroxidation extent in a control sample in which in the preparation of liposomes, an antioxidant such as (±)-α-tocopherol, was added. Interestingly the addition of 0.05% w/w (±)-α-tocopherol in the system reduced the peroxidation of linoleic acid of 50.5%. Blank samples produced negligible lipid peroxidation.

## Conclusions

In this work we demonstrated that the series of compounds herein reported, and in particular compound **9d**, are endowed with a broad-spectrum activity against a panel of seven different enveloped viruses including a HSV-2 acyclovir resistant strain, due to an irreversible mechanism that impairs viral entry into the host cell. The selective activity on enveloped viruses and the lipid peroxidation capability of the compound suggest a mechanism of action on the viral envelope that is affecting viral and cell membrane fusion. These results open interesting possible applications for these molecules as antivirals or as components of microbicidal preparations for topical use. This is of particular interest if we consider that all the recent epidemics of emerging and re-emerging viruses are caused by enveloped viruses such as Ebola virus, Lassa virus, Zika virus, SARS and MERS Coronavirus and avian flu [3], without considering the threatening infections of Hendra and Nipah viruses and the emergence of novel mutant strains. Finally, the activity of the reported compounds against HIV [6], HSV-1, HSV-2 and ZIKV, which can be all transmitted through sexual activity, suggest a possible use of this class of compounds as vaginal microbicide components.

## Materials and methods

### Chemistry

#### General information

All commercially available chemicals were used as purchased. Anhydrous reactions were run under a positive pressure of dry N_2_. Thin-layer chromatography (TLC) was carried out using Merck TLC plates: silica gel 60 F254. Chromatographic purifications were performed on columns packed with Merck 60 silica gel, 23–400 mesh, for the flash technique. ^1^H and ^13^C NMR spectra were recorded at 400 MHz on a Bruker Avance DPX400 spectrometer. Melting points were measured using a Gallenkamp melting point apparatus and are uncorrected. Microwave irradiation experiments were conducted using a CEM Discover Synthesis Unit (CEM Corp., Matthews, NC, USA). The instrument consists of a continuous focused microwave power delivery system with operator-selectable power output from 0 to 300 W. The temperature of the contents of the vessel was monitored with a calibrated IR temperature control mounted under the reaction vessel. All experiments were performed using a stirring option, whereby the contents of the vessel were stirred by a rotating magnetic plate located below the floor of the microwave cavity and a teflon-coated magnetic stir bar in the vessel.

#### General procedure for the synthesis of aldehydes 4 and 5

Methyl-4-iodosalycilate **1** or 4-Chloro-3-iodobenzotrifluoride **2** (1.00 mmol) and 5-formyl-2-furan boronic acid **3** were dissolved in 10 mL of DMF and 15 mL of EtOH. The reaction mixture was stirred for 10 min under N_2_, then Pd(PPh_3_)_2_Cl_2_ (0.10 mmol) was added and finally Na_2_CO_3_ 2M (6.00 mmol). The reaction mixture (light-orange) was stirred under N_2_ at room temperature. After 1h the reaction went to completion and was quenched with H_2_O and 2N HCl; then EtOAc was added and the mixture was stirred until the two layers became clear. The aqueous layer was extracted three times with EtOAc, then the organic phase was washed several times with H_2_O and brine, dried over Na_2_SO_4_, filtered and evaporated under reduced pressure.

#### 5-(2-chloro-5-(trifluoromethyl)phenyl)furan-2-carbaldehyde (4)

The crude product was purified by flash chromatography using PE/EtOAc = 4:1 as eluent to yield the wished product **4** as a brown solid (yield: 20%); mp = 157°C (decomposition); ^1^H NMR (400 MHz, CDCl_3_) δ 9.73 (s, 1H), 8.23 (s, 1H), 7.76–7.45 (m, 2H), 7.42–7.28 (m, 2H)ppm. **MS (ESI):** m/z 273.0 [M-H]^-^;

#### Methyl 4-(5-formylfuran-2-yl)-2-hydroxybenzoate (5)

The crude product was purified by flash chromatography using PE/EtOAc = 4:1 as eluent to yield the wished product **5** as a light orange solid (yield: 96%); mp = 150°C (decomposition); ^1^H NMR (CDCl_3_, 400 MHz): ð = 10.84 (s, 1H), 9.69 (s, 1H), 7.91–7.88 (d, 1H, *J =* 12 Hz), 7.40–7.39 (d, 1H, *J =* 4 Hz), 7.35–7.32 (m, 3H), 6.94–6.93 (d, 1H, *J =* 4Hz), 3.97 (s, 3H); ^13^C (CDCl_3_, 100 MHz): ð = 177.47, 161.69, 157.37, 152.53, 135.17, 130.58,122.56, 115.74, 113.73, 112.82, 109.84, 52.40, 29.59; **MS (ESI):** m/z 245.0 [M-H]^-^;

#### 4-(5-Formylfuran-2-yl)-2-hydroxybenzoic acid (6)

Compound **5** was dissolved in 25 mL of CH_3_OH, then a solution of NaOH 1M (5.00 mmol) was added dropwise, after the reaction mixture was heated at reflux. The reaction mixture was stirred overnight until completion (TLC). Organic solvent was removed under reduced pressure, then some water was added and the aqueous layer was extracted three times with Et_2_O; the aqueous layer was then acidified to pH 1 with HCl 6N and a precipitated appeared. (**6**) was obtained as a brown-red solid (yield: 95%); mp = 230°C (decomposition); ^1^H NMR (DMSO, 400 MHz): ð = 9.63 (s, 1H), 7.88–7.86 (d, 1H, *J =* 8 Hz), 7.66–7.65 (d, 1H, *J =* 4 Hz), 7.45–7.39 (m, 3H); ^13^C NMR (DMSO, 100 MHz): ð = 178.72, 171.68, 161.69, 156.82, 152.63, 135.11, 131.72, 125.14, 116.02, 114.06, 113.29, 111.53; **MS (ESI):** m/z 231.0 [M-H]^-^;

#### General procedure for the synthesis of final compounds 9a-e

To a solution of bis(carboxymethyl)trithiocarbonate (0.22 mmol) in DME (1.0 mL) were added TEA (0.22 mmol) and the opportune amine (0.22 mmol). The reaction mixture was heated at 90°C for 10 min under microwave irradiation. After this time, the aldehyde **6** (0.22 mmol) was added and the mixture was heated at 110°C for 5 min under microwave irradiation. The reaction mixture was evaporated to dryness, then MeOH and a drop of HCl 2N were added; the final rhodanine derivatives were obtained as a pure precipitate, isolated by filtration, washed with water and hexane, and finally dried under high vacuum.

#### (Z)-4-(5-((3-(2-(1H-Indol-3-yl)ethyl)-4-oxo-2-thioxothiazolidin-5- ylidene)methyl)furan-2-yl)-2-hydroxybenzoic acid (9a)

(Yield: 55%); Yellow-brown solid; mp = 254°C (decomposition); ^1^ H NMR: ((CD_3_)_2_CO, 400 MHz) δ = 8.00–7.98 (d, 1H, *J* = 8.4 Hz), 7.76–7.74 (d, 1H, *J* = 8 Hz), 7.64 (s, 1H), 7.47–7.33 (m, 5H), 7.22 (s, 1H), 7.11–7.02 (m, 2H) 4.41–4.37 (m, 2H), 3.19–3.15 (m, 2H); ^13^C NMR (DMSO, 100 MHz): δ = 194.15, 171.77, 166.77, 161.81, 156.64, 150.49, 136.64, 134.91, 131.77, 127.45, 123.59, 123.10, 121.47, 120.25, 118.86, 118.53, 118.33, 115.53, 113.52, 113.09, 112.40, 111.90, 110.38, 45.23, 22.82; **MS (ESI):** m/z 489.0 [M-H]^-^;

#### (Z)-4-(5-((3-(4-bromophenethyl)-4-oxo-2-thio-oxothiazolidin-5-ylidene)methyl)furan-2-yl)-2-hydroxybenzoic acid (9b)

(Yield: 89%); Brown solid. Mp = 275°C (decomposition); ^1^H NMR: (DMSO, 400 MHz) δ = 7.93–7.91 (d, 1H, J = 8.4 Hz), 7.64 (s, 1H), 7.48–7.46 (m, 3H), 7.39–7.36 (m, 3H), 7.18–7.16 (m, 2H), 4.24–4.21 (m, 2H), 2.96–2.93 (m, 2H) ppm. ^13^C NMR (DMSO, 100 MHz): δ = 194.07, 171.54, 166.68, 161.86, 156.87, 150.49, 137.55, 134.85, 131.72, 131.50, 131.34, 123.05, 120.02, 119.43, 118.48, 115.51, 114.05, 112.90, 112.53, 45.32, 31.96 ppm. **MS (ESI):** m/z 529.0 [M-H]^-^. Purity: 97.5%.

#### (Z)-2-hydroxy-4-(5-((3-(4-methoxyphenethyl)-4-oxo-2-thioxothiazolidin-5-ylidene)methyl)furan-2-yl)benzoic acid (9c)

(Yield: 79%); Yellow solid. Mp = 265°C (decomposition); ^1^H NMR: (DMSO, 400 MHz) δ = 7.90–7.88 (d, 1H, *J =* 8.0 Hz), 7.60 (s, 1H), 7.45–7.44 (d, 1H, *J =* 3.6 Hz), 7.36–7.33 (m, 3H), 7.13–7.10 (d, 2H, *J =* 8.4 Hz), 6.85–6.83 (d, 2H, *J =* 8.4 Hz), 4.18–4.14 (m, 2H), 3.70 (s, 3H), 2.89–2.85 (m, 2H) ppm. ^13^C NMR (DMSO, 100 MHz): δ = 193.99, 171.74, 166.65, 161.81, 158.40, 156.68, 150.46, 134.89, 131.76, 130.05, 129.82, 123.13, 120.00, 118.39, 115.53, 114.33, 113.60, 113.06, 112.42, 55.37, 45.85, 31.67 ppm. **MS (ESI):** m/z 481.0 [M-H]^-^. HPLC: purity: 98.5%.

#### (Z)-2-hydroxy-4-(5-((3-(4-isopropylphenethyl)-4-oxo-2-thioxothiazolidin-5-ylidene)methyl)furan-2-yl)benzoic acid (9d)

(Yield: 87%); Orange solid. Mp = 259°C (decomposition); 1H NMR: (DMSO, 400 MHz) δ = 7.89–7.87 (d, 1H, *J* = 8.0 Hz), 7.59 (s, 1H), 7.43–7.40 (m, 2H), 7.34–7.31 (m, 2H), 7.15–7.12 (m, 4H), 4.18–4.14 (m, 2H), 2.89–2.85 (m, 2H), 1.39 (s, 1H), 1.17–1.15 (m, 6H) ppm. ^13^C NMR (DMSO, 100 MHz): δ = 193.97, 171.77, 166.62, 161.83, 156.69, 150.45, 147.10, 135.29, 134.86, 131.72, 128.91, 126.83, 125.16, 123.13, 120.02, 118.36, 115.96, 115.51, 113.58, 113.03, 112.41, 45.68, 33.42, 32.18, 24.26 ppm. **MS (ESI):** m/z 493.0 [M-1]^-^. HPLC: purity: 98.0%.

#### (Z)-5-((5-(2-chloro-5-(trifluoromethyl)phenyl)furan-2-yl)methylene)-3-(4-fluorobenzyl)-2-thioxothiazolidin-4-one 7 (9e)

(Yield: 20%); Orange solid. Mp = 259°C (decomposition); ^1^H NMR (400 MHz, CDCl_3_) δ 7.62 (d, *J* = 8.4 Hz, 1H), 7.56–7.50 (m, 2H), 7.40 (dd, *J* = 10.3, 6.0 Hz, 3H), 7.31–7.23 (m, 4H)ppm. ^13^C NMR (DMSO, 100 MHz): δ = 193.97, 171.77, 166.62, 161.83, 156.69, 150.45, 147.10, 135.29, 134.86, 131.72, 128.91, 126.83, 125.16, 123.13, 120.02, 118.36, 115.96, 115.51, 113.58, 113.03, 112.41, 45.68, 33.42, 32.18, 24.26 ppm. **MS (ESI):** m/z 496.0 [M-H]^-^. HPLC: purity: 98.0%.

#### General procedure for the synthesis of compounds 11a and 11b

Benzoylchloride **10** (4.31 mmol, 1.00 eq) was dissolved in 3 mL of acetone. To this, NH_4_SCN (5,17 mmol, 1.2 eq) was added in one portion and the mixture irradiated at 60°C for 15 min at the microwave. After this time, the opportune amine (1.00 eq) respectively benzylamine for **8a**, and phenethylamine for **8b**, was added, and the mixture was irradiated for further 15 min at the microwave. The resulting suspension was filtered, and the precipitate washed with H_2_O and CH_3_OH, to furnish pure **8a** and **8b** as white solids.

#### N-(benzylcarbamothioyl)benzamide (11a)

Yield 50% white solid. **^1^H NMR**: (400 MHz CDCl_3_) *δ (ppm)* 11.05 (bs, 1H), 9.27 (bs, 1H), 7.80 (d, 2H, *J =* 8.4 Hz), 7.57 (t, 1H, *J =* 14.8 Hz), 7.45 (t, 2H, *J =* 15.2 Hz), 7.39–7.30 (m, 5H), 4.91 (d, 2H, *J =* 5.2 Hz)ppm. **MS:** (ESI) *m/z* 268.9 [M-H]^-^

#### N-(phenethylcarbamothioyl)benzamide (11b)

Yield 47% white solid. ^**1**^**H NMR:** (400 MHz CDCl_3_) *δ (ppm)* 10.78 (bs, 1H), 9.09 (bs, 1H), 7.80 (d, 2H, *J =* 7.2 Hz), 7.60 (t, 1H, *J =* 14.8 Hz), 7.48 (t, 2H, *J =* 15.6 Hz), 7.36–7.23 (m, 5H), 3.95 (q, 2H, *J =* 20.4 Hz), 3.03 (t, 2H, *J =* 14.4 Hz)ppm. **MS:** (ESI) *m/z* 282.9 [M-H]^-^

#### General procedure for the synthesis of compounds 12a and 12b

The opportune thioureido derivative (**11a** or **11b**) (1.61 mmol) was dissolved in 10 mL of hydrazine monohydrate. The solution was stirred at RT for 3 h. Then 5 mL of fuming hydrochloric acid were added and the mixture was extracted with CH_2_Cl_2_ (3 x 15 mL). The organic phase was washed with Na_2_CO_3_ (aq. sol.) (2 x 50 mL), and dried over Na_2_SO_4_. The solvent was removed at reduced pressure and the corresponding residue purified by flash chromatography (AcOEt/CH_2_Cl_2_ 1:1).

#### 1-benzylthiourea (12a)

Yield 80% white solid. ^**1**^**H NMR:** (400 MHz DMSO-*d*_6_) *δ* 7.94 (bs, 1H), 7.33–7.21 (m, 5H), 7.03 (bs, 2H), 4.60 (s, 2H)ppm; **MS:** (ESI) *m/z* 166.9 [M+H]^+^;

#### 1-phenethylthiourea (12b)

Yield 95% white solid.^**1**^**H NMR:** (400 MHz DMSO-*d*_6_) *δ* 7.33 (t, 2H, *J =* 14.4 Hz), 7.26 (t, 1H, 6.8 Hz), 7.21 (d, 2H, *J =* 7.2 Hz), 5.68 (bs, 2 H), 2,91 (t, 2H, *J =* 14.0 Hz)ppm. **MS:** (ESI) *m/z* 178.9 [M-H]^-^

#### General procedure for the synthesis of compounds 14a and 14b

Sodium metal (3,61 mmol, 3 eq) was dissolved in anhydrous EtOH (10 mL), then the opportune thiourea compound (**12a** or **12b**) (1.20 mmol) and diethylmalonate **13** (2.41 mmol) were added subsequently, and the mixture was refluxed under Argon atmosphere. The solvent was removed at reduced pressure and the corresponding residue was solubilized in water. The mixture was acidified at pH 2 with HCl 1N and filtered. The residue was then purified by flash chromatography (CH_2_Cl_2_/CH_3_OH 8:2).

#### 1-benzyl-2-thioxodihydropyrimidine-4,6(1H,5H)-dione (14a)

Yield 80% red solid. ^**1**^**H NMR:** (400 MHz DMSO-*d*_6_) *δ* 10.54 (bs, 1H), 7.23–7.15 (m, 5H), 5.39 (s, 2H), 4.30 (s, 1 H)ppm. **MS:** (ESI) *m/z* 232.9 [M-H]^-^

#### 1-phenethyl-2-thioxodihydropyrimidine-4,6(1H,5H)-dione (14b)

Yield 80% white solid. ^**1**^**H NMR:** (400 MHz DMSO-*d*_6_) *δ (ppm)* 10.76 (bs, 1H), 7.29–7.19 (m, 5H), 4.33 (t, 2H, *J =* 16.0), 3.14 (s, 1 H), 2.83 (t, 2H, *J =* 16.0)ppm. **MS:** (ESI) *m/z* 246.9 [M-H]^-^

#### General procedure for the synthesis of compounds 15a and 15b

Compound **6** (0,22 mmol, 1 eq) and compound **13a** or **13b** (0.22 mmol, 1 eq) were suspended in EtOH (6 mL); to this, 3 drops of HCl (conc.) were added and the mixture was stirred at 70°C overnight. After this time 3 mL of HCl were added and the resulting precipitate was filtered at reduced pressure to give a solid that was washed with H_2_O, CH_3_OH and hexane.

#### (Z)-4-(5-((1-benzyl-4,6-dioxo-2-thioxotetrahydropyrimidin-5(2H)-ylidene)methyl)furan-2-yl)-2-hydroxybenzoic acid (15a)

(Yield 53%) red solid, ^**1**^**H NMR:** (400 MHz DMSO-*d6*) *δ* 8.57 (d, 1H, 3,6), 8.12 (s, 1H), 7.84 (d, 1H, *J =* 8.0 Hz), 7.56 (d, 1H, *J =* 3.6 Hz), 7.45–7.42 (m, 2H), 7-29-7.20 (m, 5H), 5.52 (s, 2H)ppm, ^**13**^**C NMR:** (101 MHz DMSO-*d6*) *δ* 179.42, 171.68, 162.05, 161.72, 160.30, 159.71, 158.73, 151.34, 138.28, 136.86, 134.45, 131.61, 130.83, 128.57, 127.31, 116.43, 114.46, 113.68, 113.38, 113.26, 49.55 ppm. **MS:** (ESI) *m/z* 446.7 [M-H]^-^

#### (Z)-4-(5-((4,6-dioxo-1-phenethyl-2-thioxotetrahydropyrimidin-5(2H)-ylidene)methyl)furan-2-yl)-2-hydroxybenzoic acid (15b)

(Yield 80%) brown solid, ^**1**^**H NMR:** (400 MHz DMSO-*d6*) *δ* 8.59 (s, 1H), 8.14 (s, 1H), 7.88 (d, 1H, *J* = 8.0), 7.62 (d, 1H, *J* = 3.6), 7.52–7.49 (m, 2H), 7-33-7.22 (m, 5H), 4.45 (m, 2H, *J* = 22.0 Hz), 2.91 (q, 2H, 22.4)ppm; ^**13**^**C NMR:** (101 MHz DMSO-*d6*) *δ* 178.97, 171.69, 161.79, 160.38, 159.64, 158.81, 151.34, 151.19, 138.84, 137.63, 134.53, 131.70, 130.72, 128.96, 126.86, 116.47, 114.63, 114.46, 113.73, 48.96, 32.96 ppm, **MS:** (ESI) *m/z* 460.8 [M-H]^-^

### Cells

African green monkey fibroblastoid kidney cells (Vero, ATCC CCL-81), human epithelial cells (Hep-2) (ATCC CCL-23) African green monkey kidney epithelial (MA-104) cells (ATCC CRL-2378.1), Madin Darby Canine Kidney (MDCK ATCC CCL-34) were grown as monolayers in Eagle's minimal essential medium (MEM) (Gibco/BRL, Gaithersburg, MD) supplemented with 10% heat inactivated fetal calf serum (FCS) and 1% antibiotic-antimycotic solution (Zell Shield, Minerva Biolabs GmbH, Berlin, Germany). Low-passage human embryonic lung fibroblasts (HELFs) were grown as monolayers in Eagle's minimal essential medium (Gibco-BRL) in the same conditions as described above with the addition of 1 mM sodium pyruvate. Human epithelial adenocarcinoma HeLa cells (ATCC CL-2^TM^) were propagated in Dulbecco’s modified Eagle’s medium (DMEM) supplemented as described above. The 293TT cell line, derived from human embryonic kidney cells transformed with the simian virus 40 (SV40) large T antigen, was cultured as monolayer in medium in DMEM supplemented with Glutamax-I (Invitrogen, Carlsbad, CA) and nonessential aminoacids.

### Viruses

Clinical isolates of HSV-1 and HSV-2 were kindly provided by Prof. M. Pistello, University of Pisa, Italy. HSV-1 and HSV-2 strains were propagated and titrated by plaque assay on Vero cells. A HSV-2 strain with phenotypic resistance to acyclovir was generated by serial passage in the presence of increasing concentrations of acyclovir and tested for acyclovir resistance with dose-response inhibition assay with an EC50 of 319 μM, as previously described [17]. HCMV strain Towne was kindly provided by Prof. W. Brune, Heinrich Pette Institut, Hamburg, Germany; it was propagated and titrated by plaque assay on HELF cells. RSV strain A2 (ATCC VR-1540) was propagated in Hep-2 and titrated by the indirect immunoperoxidase staining procedure using an RSV monoclonal antibody (Ab35958; Abcam, Cambridge, United Kingdom), as described previously [15]. Human rotavirus strain Wa (ATCC VR-2018) was activated with 5 mg/mL porcine pancreatic trypsin type IX (Sigma, St. Louis, Mo.) for 30 min at 37°C and propagated in MA104 cells using MEM containing 0.5 mg trypsin per mL, as described previously [18]. VSV (ATCC VR-1238) was propagated on Vero cells and titrated by plaque assay. Zika virus (PRVABC59) was kindly provided by Marco Alves and was propagated and titrated by plaque assay on Vero cells. IAV H1N1 isolated from a clinical specimen was propagated and titrated on MDCK. Adenovirus 5 encoding GFP (GFP-Ad5), with a E1/E3 deletion, was purchased from Vector Biolabs (Philadelphia, PA, USA). HPV-16 pseudovirions were produced with 293TT as previously described [19] and their concentration was assessed on Hela cells. Virus stocks were maintained at -80°C.

### Cell viability

Cell viability was measured using the MTS [3- (4,5-dimethylthiazol-2-yl)-5- (3 carboxy methoxy phenyl)-2-(4-sulfophenyl)-2H-tetrazolium] assay. Cell cultures were seeded in 96-well plates and were incubated with different concentrations of compounds in duplicate under the same experimental conditions described for the antiviral assays, (i.e. if the incubation time of the compound on cells was carried for 2 hours and then the evaluation of the antiviral activity was done 72h later, the same timing was used for the cytotoxicity assays, in order to exclude any toxicity effect in the antiviral evaluation). Cell viability was determined using the CellTiter 96 Proliferation Assay Kit (Promega, Madison, WI, USA) according to the manufacturer's instructions. Absorbances were measured using a Microplate Reader (Model 680, BIORAD) at 490 nm. The effect on cell viability at different concentrations of the compound was expressed as a percentage, by comparing absorbances of treated cells with those of cells incubated with culture medium and equal volumes of vehicle. The 50% cytotoxic concentrations (CC_50_) and 95% confidence intervals (CIs) were determined using Prism software (Graph-Pad Software, San Diego, CA). For compound 9d a continuous incubation of 1, 2, 3 and 4 days on Vero cells was carried in order to evaluate the toxicity after long exposure the results are shown in S1 Fig and Fig 3.

### HSV, VSV and ZIKV inhibition assays

The antiviral effect on HSV and VSV and ZIKV infection was evaluated by plaquing efficiency assay. Vero cells were pre-plated 24 h in advance in 24-well plates at a density of 10^5^ cells. Increasing concentrations of compounds were mixed with HSV-2 (MOI 0.001 pfu/cell) or HSV-2 acyclovir resistant (MOI 0.001) or HSV-1 (MOI 0.0005) or VSV (MOI 0.005) or ZIKV (MOI 0.005) and incubated for 1 hour at 37°C. The mixtures were subsequently added to the cells, which were then incubated at 37°C for 2 h. The virus inoculum was then removed and the cells washed and overlaid with a medium containing 1.2% methylcellulose (Sigma). After further incubation at 37°C for 24 h (HSV-2 and VSV) or 48 h (HSV-1) or 72h (ZIKV), cells were fixed and stained with 0.1% crystal violet in 20% ethanol and viral plaques counted. The effective concentration producing 50% reduction in plaque formation (EC_50_) was determined using Prism software by comparing drug-treated with wells treated with medium and solvent. The selectivity index (SI) was calculated by dividing the CC_50_ by the EC_50_ value.

#### HCMV inhibition assays

HELF cells were pre-plated in a 96-well plate. The following day increasing concentrations of compounds were mixed with HCMV (MOI 0.005) and incubated for 1 h at 37°C. The mixtures were subsequently added to the cells, which were then incubated at 37°C for 3 h; monolayers were then washed and overlaid with 1.2% methylcellulose medium supplemented with 3% FCS and 1mM sodium pyruvate. After five days incubation, cells were observed under an inverted Zeiss LSM510 fluorescence microscope (Zeiss, Oberkochen, Germany) and the percentages of infection were calculated by comparing GFP positive cells in treated and untreated wells.

#### RSV inhibition assays

HeP-2 cells were pre-plated in a 96-well plate. The following day increasing concentrations of compounds were mixed with RSV (MOI 0.005) and incubated for 1 h at 37°C. The mixtures were subsequently added to the cells, which were then incubated at 37°C for 3 h; monolayers were then washed and overlaid with 1.2% methylcellulose medium. 72 h later cells were fixed and subjected to specific immunostaining using an RSV monoclonal antibody (Ab35958; Abcam, Cambridge, United Kingdom) in order to visualize syncytia. Percentages of infection were calculated by comparing numbers of syncytia in treated and untreated wells.

#### HRoV inhibition assays

MA104 cell were plated in 96-well trays. The following day virus infectivity was activated by adding 5 μg porcine trypsin (Sigma)/mL for 30 min at 37°C. Increasing concentrations of compounds were mixed with HRoV (MOI 0.02) and incubated for 1 h at 37°C. The mixtures were subsequently added to the cells, which were then incubated at 37°C for 1 h; monolayers were then washed and overlaid with medium. After 16 h, cells were fixed with cold acetone–methanol and viral titers were determined by indirect immunostaining using the monoclonal antibody mab-0036 (specific for human 41 kDa inner capsid protein–VP6 –of rotavirus) purchased from Covalab (Villeurbanne, France) and the UltraTech HRP Streptavidin-Biotin Detection System (Beckman Colter).

#### HPV-16 and Ad5 inhibition assays

HeLa cells were plated in 96-well plates. The following day, increasing concentrations of compounds were mixed with HPV-16 (approximately 1 ng/mL L1) or Ad5 (MOI 0.02) and incubated for 1 h at 37°C. The mixtures were subsequently added to the cells, which were then incubated at 37°C for 72 h. The GFP-expressing infected cells were observed under an inverted Zeiss LSM510 fluorescence microscope (Zeiss, Oberkochen, Germany) and the percentages of infection were calculated by comparing GFP positive cells in treated and untreated wells.

#### IAV H1N1 inhibition assays

MDCK cells were pre-plated in 96-well plates. The following day, increasing concentrations of compounds were mixed with IAV (MOI 0.05) and incubated for 1 h at 37°C. The mixtures were subsequently added to the cells for 1 h at 37°C, after a washout cells were overlaid with medium for 16 h at 37°C. Cells were then fixed and subjected to specific immunostaining using a Flu A monoclonal antibody (Merck 5001) in order to visualize infected cells. Percentages of infection were calculated by comparing numbers of infected cells in treated and untreated wells.

### Mechanism of action study

Vero cells were subjected to different assays:

Pretreatment: cells were pretreated for 2 h at 37°C with increasing concentrations of compounds, the inocula were then removed, cells washed and HSV-2 was added on cells for 2 h at 37°C. Subsequently the same protocol described above was followed.

During infection: Cells were subjected to the same experiment described above without the pre-incubation between compounds and virus. Post treatment: Cells were infected with HSV-2 (MOI 0.01) for 2 h at 37°C, the viral inoculum was removed and cultures were exposed to different compound concentrations and incubated until control cultures displayed extensive cytopathology. Supernatants and cells were harvested and cell-free virus infectivity titers were determined in duplicate by plaque assay in Vero cell monolayers. Percent inhibition was determined by comparing the titer measured in the presence of the compounds to that measured in untreated wells.

Binding: Cells were plated in 96 well plates. The following day 10 μM of compound and HSV-2 (MOI 10) were incubated for 1 h at 37°C and subsequently added on cells for 2 h at 4°C. Cells were then fixed with 4% paraformaldehyde, air dried, and blocked with 5% bovine serum albumin (BSA) in phosphate-buffered saline (PBS)–Tween. Bound virus was detected using the polyclonal HSV-2 antibody (Dako, Denmark) (diluted 1:250) incubated for 1 h at room temperature, washed three times with PBS-Tween, and incubated for 1 h at 37°C with anti-rabbit conjugated to horseradish peroxidase (1:500). At the end of incubation, plates were washed three times with PBS-Tween, ABTS [2,2-azinobis(3-ethyl benz thiazoline sulfonic acid)] substrate was added for 30 min (Thermo Scientific, Rockford, IL) and the absorbance at 405 nm was read.

Entry: Cells were plated in 96 well plates. The following day 10 μM of compound and HSV-2 (MOI 10) were incubated for 1 h at 37°C and subsequently added on cells for 1 h at 4°C. Cells were then washed and shifted at 37°C for 1.5 h or 4 h, after they were subjected to a wash with acidic glycine to inactivate unentered virus. Cells were then fixed with 4% paraformaldehyde, air dried and permeabilized with PBS-TRITON (0.5%) and then the same protocol described for binding was followed.

Virucidal assay: Approximately 10^5^ PFU of HSV2 plus 10 μM or different concentration of compound were added to MEM and mixed in a total volume of 100 μL. The virus-compound mixtures were incubated for different times at 37°C then diluted serially to the non-inhibitory concentration of test compound on cells or in tubes for additional 1, 2, 3 or 4 hours; the residual viral infectivity was determined by viral plaque assay.

### Immunofluorescence

Cells were plated on coverslips and the following day the binding or entry protocol described above was applied. Cells were than fixed with 4% paraformaldehyde, air dried and for entry protocol permeabilized with PBS-TRITON. Blocking was performed with PBS-BSA 1% for 1 h at room temperature and subsequently HSV-2 polyclonal antibody (1:250) was added on cells, after 1 h at 37°C the inoculum was removed and coverslips were washed with PBS-TWEEN for 3 times. Rhodamine conjugated anti-rabbit (Santa Cruz) was then added on cells for 1 h at 37°C following 3 washes coverslips were mounted on microscope glasses and observed with Zeiss LSM510 fluorescence microscope (Zeiss, Oberkochen, Germany) and images were acquired.

### qPCR

Cells subjected to binding assays were then lysed and subjected to total DNA/RNA extraction and subsequently subjected to qPCR using as primers 5’- CCGTCAGCACCTTCATCGA -3’ and 5’-CGCTGGACCTCCGTGTAGTC -3’ and as probe 5’-FAM CCACGAGATCAAGGACAGCGGCC-TAMRA for 40 cycles at 94°C for 15” followed by 60°C for 60”. The results were normalized according to values of rnasep and the % of bound virus were measured comparing the Δct of treated wells to wells treated with equal volume of solvent.

### In vitro determination of the compound peroxidation capability

To evaluate the oxidation capability of **9d** an *in vitro* lipoperoxidation test was carried out. In lipid peroxidation, free radicals attack double bonds of polyunsaturated fatty acids forming lipid hydroperoxides, which undergo homolytic scission to form a variety of cytotoxic compounds, such as aldehydes. This oxidative stress plays an important role in damaging membrane lipids. According to previous literature, the potential oxidative effect of **9d** was evaluated toward the oxidation of linoleic acid, a carboxylic acid with two double bonds as a model lipid substrate. The lipoperoxidation of linoleic acid, incorporated in dipalmitoylphosphatidylcholine (DPPC) liposomes was determined using the TBA assay [20, 21]. DPPC liposomes were prepared with the thin film evaporation method and used to mimic a phospholipid bilayer, and to dissolve linoleic acid. TBA assay, commonly used as an index of lipid peroxidation, is based on the reactivity of malondialdehyde (MDA), a colourless end-product of degradation, with 2-thiobarbituric acid (TBA) to produce a pink adduct (TBA-MDA-TBA) that absorbs at 535 nm. MDA is indeed one of the final products of polyunsaturated fatty acids peroxidation in the cells and is generally considered an indicator of lipid peroxidation. MDA was detected spectrophotometrically according to the method described by Bay et al. with the following modifications [22].

For the TBA assay,**9d** compound was dissolved in DMSO at the concentration of 10 μM. Then, 100 μl of the **9d** DMSO solution were incubated at 37°C for 30 minutes with a 0.5% linoleic acid containing liposome aqueous dispersion. Blank samples were prepared incubating the liposome aqueous dispersion either with saline solution (0.9% NaCl aqueous solution) or DMSO in the absence of **9d**.

After incubation, a volume of the blank and **9d** samples (0.2 mL) were withdrawn and introduced in a glass tube closed with a screw cap and added with 0.1 mL of water, 0.2 mL of 4% w/w SDS, 1.5 mL of 1.0% w/w phosphoric acid and 1.0 mL of 0.6% w/w TBA. The mixture was stirred and heated in water bath at 95–100°C for 45 min to favor the formation of the complex. After cooling in an ice bath, 4.0 mL of 1-butanol were added to each tube and the TBA-MDA-TBA complex was extracted upon stirring and centrifugation. The organic supernatant was evaluated by spectrophotometry. The calibration curve of TBA-MDA-TBA complex was obtained using a MDA precursor 1,1,3,3-tetraethoxypropane. MDA can be obtained by acid hydrolysis from 1,1,3,3-tetraethoxypropane in an equimolecular reaction. For this purpose, standard solutions of 1,1,3,3-tetraethoxypropane in SDS (4% w/w) within the concentration range 5–250 μM were prepared. The solutions were subjected to TBA assay and analyzed at spectrophotometer. The final concentration of MDA derived from the reaction of linoleic acid, calculated exploiting the calibration curve, was expressed as micromoles of MDA per mg of lipid substrate. To further confirm the oxidant activity of **9d** the peroxidation experiment was also carried out using a control sample containing 0.05% w/w of (±)-α-tocopherol, molecule with antioxidant properties, added during the preparation of liposomes. MDA formation was monitored in the same conditions above reported. The results are mean and SD of 3 independent experiments.

### Data analysis

Results are presented as the mean values from 2 to 6 independent experiments, in case of 2 independent experiments results are expressed ± square root of the sum of squares, in other cases as ± SD. The EC_50_ values for inhibition curves were calculated by regression analysis using the software GraphPad Prism (GraphPad Software, San Diego, California, U.S.A.) by fitting a variable slope-sigmoidal dose–response curve. For binding and entry assays the significance was analyzed with a One-way Anova followed by a Bonferroni.

## Supporting information

S1 Fig (A) Viability was evaluated after 1, 2, 3 h of exposure 24 h post-treatment. (B) Viability was evaluated after 24, 48, 72, 96 h of exposure. % of viability were calculated through a ratio between absorbance of wells treated with compound 9d to wells treated with equal volume of DMSO. Results are mean and square root of the sum of squares. n = 2.
(DOCX)

S2 Fig Virucidal activity was evaluated through incubation with 9d compound for 1h followed by dilution in drug free medium for 1, 2, 3 or 4 h and subsequent addition on cells. Results are mean and SD. n = 3.
(DOCX)
