# Supplementary material for: Novel broad spectrum virucidal molecules against enveloped viruses
Source: PLoS One. 2018 Dec 7;13(12):e0208333. doi: 10.1371/journal.pone.0208333 (PMC6285983; doi:10.1371/journal.pone.0208333)
Supplement: S1 Fig — (A) Viability was evaluated after 1, 2, 3 h of exposure 24 h post-treatment. (B) Viability was evaluated after 24, 48, 72, 96 h of exposure. % of viability were calculated through a ratio between absorbance of wells treated with compound 9d to wells treated with equal volume of DMSO. Results are mean and square root of the sum of squares. n = 2. (DOCX) [file pone.0208333.s001.docx]

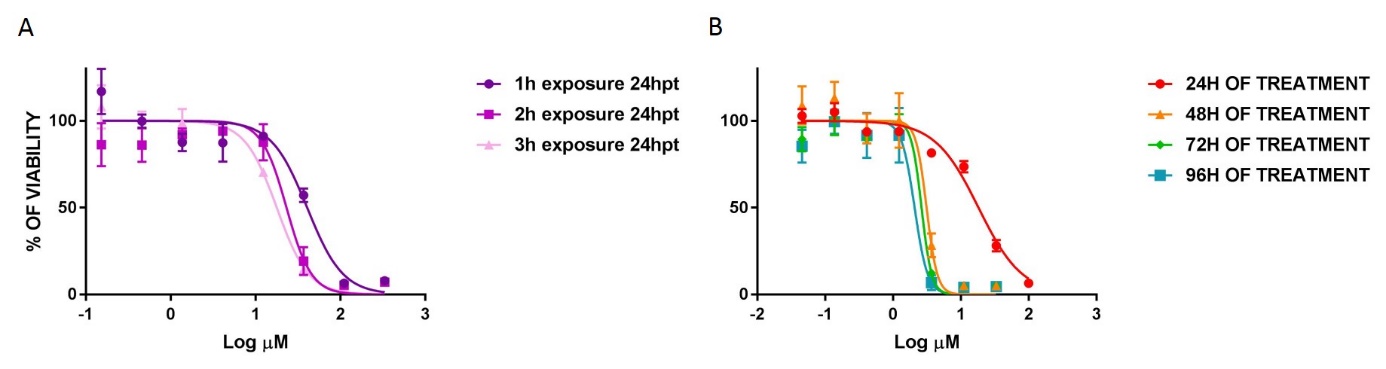


**S1 Fig.** (A) Viability was evaluated after 1, 2, 3 h of exposure 24 h post-treatment. (B) Viability was evaluated after 24, 48, 72, 96 h of exposure. % of viability were calculated through a ratio between absorbance of wells treated with compound 9d to wells treated with equal volume of DMSO. Results are mean and square root of the sum of squares. n=2.
