# Supplementary material for: Novel broad spectrum virucidal molecules against enveloped viruses
Source: PLoS One. 2018 Dec 7;13(12):e0208333. doi: 10.1371/journal.pone.0208333 (PMC6285983; doi:10.1371/journal.pone.0208333)
Supplement: S2 Fig — Results are mean and SD. n = 3. (DOCX) [file pone.0208333.s002.docx]

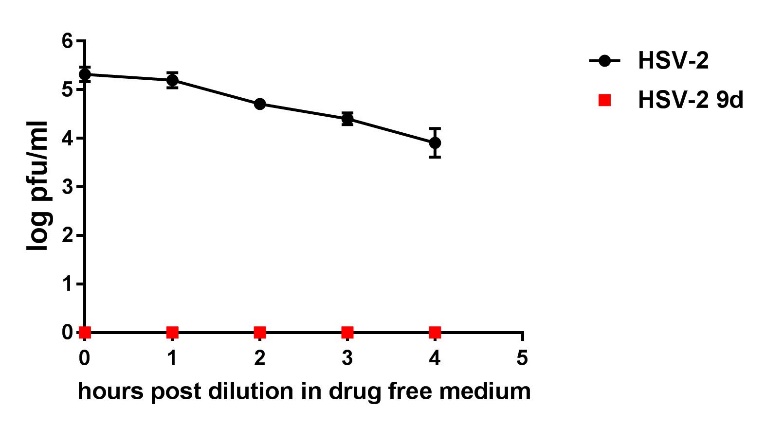


**S2 Fig.** Virucidal activity was evaluated through incubation with 9d compound for 1h followed by dilution in drug free medium for 1, 2, 3 or 4 h and subsequent addition on cells. Results are mean and SD. n=3.
